# Molecular Genetics and Complex Inheritance of Congenital Heart Disease

**DOI:** 10.3390/genes12071020

**Published:** 2021-06-30

**Authors:** Nicholas S. Diab, Syndi Barish, Weilai Dong, Shujuan Zhao, Garrett Allington, Xiaobing Yu, Kristopher T. Kahle, Martina Brueckner, Sheng Chih Jin

**Affiliations:** 1 Department of Genetics, Yale School of Medicine, New Haven, CT 06510, USA; nicholas.diab@yale.edu (N.S.D.); syndi.barish@yale.edu (S.B.); weilai.dong@yale.edu (W.D.); 2 Laboratory of Human Genetics and Genomics, The Rockefeller University, New York, NY 10065, USA; 3 Department of Genetics, School of Medicine, Washington University, St. Louis, MO 63110, USA; shujuanzhao@wustl.edu (S.Z.); xiaobing@wustl.edu (X.Y.); 4 Department of Pathology, Yale School of Medicine, New Haven, CT 06510, USA; garrett.allington@yale.edu; 5 Department of Computer Science & Engineering, Washington University, St. Louis, MO 63130, USA; 6 Department of Neurosurgery, Yale School of Medicine, New Haven, CT 06510, USA; kristopher.kahle@yale.edu; 7 Department of Pediatrics, Yale School of Medicine, New Haven, CT 06510, USA; 8 Department of Cellular & Molecular Physiology, Yale School of Medicine, New Haven, CT 06510, USA; 9 Department of Pediatrics, School of Medicine, Washington University, St. Louis, MO 63110, USA

**Keywords:** congenital heart disease, genetics, genomics, complex inheritance, histone marks, next-generation sequencing, genomic medicine, precision medicine, rare disease

## Abstract

Congenital heart disease (CHD) is the most common congenital malformation and the leading cause of mortality therein. Genetic etiologies contribute to an estimated 90% of CHD cases, but so far, a molecular diagnosis remains unsolved in up to 55% of patients. Copy number variations and aneuploidy account for ~23% of cases overall, and high-throughput genomic technologies have revealed additional types of genetic variation in CHD. The first CHD risk genotypes identified through high-throughput sequencing were de novo mutations, many of which occur in chromatin modifying genes. Murine models of cardiogenesis further support the damaging nature of chromatin modifying CHD mutations. Transmitted mutations have also been identified through sequencing of population scale CHD cohorts, and many transmitted mutations are enriched in cilia genes and Notch or VEGF pathway genes. While we have come a long way in identifying the causes of CHD, more work is required to end the diagnostic odyssey for all CHD families. Complex genetic explanations of CHD are emerging but will require increasingly sophisticated analysis strategies applied to very large CHD cohorts before they can come to fruition in providing molecular diagnoses to genetically unsolved patients. In this review, we discuss the genetic architecture of CHD and biological pathways involved in its pathogenesis.

## 1. Introduction

Congenital heart disease (CHD) is a collective diagnosis for structural malformations of the heart and great vessels [1]. Individual lesions within CHD have been grouped into anatomic subtypes since Thomas Peacock’s 1858 monograph *On Malformations of the Human Heart* [2], and anatomy remains the major classifying schema for CHD. Peacock’s same monograph also introduced the earliest hints that some congenital heart defects are genetic. Peacock documented a “hereditary predisposition” to CHD within affected families, and this predisposition would later be formalized conceptually as recurrence risk by studies that investigated CHD reappearance within and between generations of affected families. Recurrence studies historically offered key evidence in support of a genetic component to CHD and suggest that 90% of CHD includes a genetic contribution [3,4].

The first parent-offspring recurrence study was led by Dr. Ruth Whittemore at Yale [5]. Whittemore’s studies involving CHD mothers and their offspring revealed a 16% recurrence risk, in contrast to the 2–3% recurrence risk in siblings [5]. Whittemore also demonstrated lesion-specific recurrence rates, providing some of the earliest evidence that different CHD lesions are genetically distinct entities.

**Figure 1:**
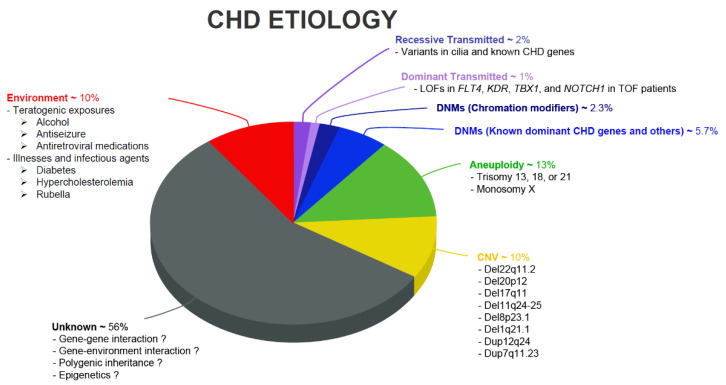
Pie chart showing various CHD etiologies and their relative disease contribution. The contribution of de novo mutations to CHD was first estimated by Zaidi et al. [8], while Jin et al. [10] provided the first model for systematic interrogation of transmitted CHD mutations. Non-genetic etiologies of CHD are reviewed elsewhere [23,24,25,26,27].

The genetics of CHD have been advanced by many groups, including large consortiums such as the Pediatric Cardiac Genomics Consortium (PCGC) [6] and the Deciphering Developmental Disorders project (DDD) [7], which have recruited very large CHD cohorts with the goal to discover a complete repertoire of genes responsible for CHD. The timing of the PCGC’s and DDD’s inception coincided with the advent of next-generation sequencing technology, opening the floodgates to scalable analysis of genetic variants in CHD [8,9,10,11,12,13,14,15,16,17,18]. Meanwhile, many other groups in the world have made equally important contributions to both the identification of novel risk loci/genes, biological pathways, and cellular/molecular processes for CHD pathogenesis [19,20,21,22]. These studies together provide a more complete picture of the role of genetic variation in CHD. Overall, the combination of large consortium efforts and work by individual laboratories has delineated the variable contributions of various types of genetic variation to CHD (Figure 1).

Despite exhaustive efforts by many groups, the causative genetic mechanisms behind CHD remain poorly understood and ~55% of CHD patients lack a genetic diagnosis (Figure 1). Recent advances in high-throughput genomic technologies, such as single-cell sequencing, transcriptomic profiling, and stem cell biology, now permit analyzing genomic DNA, RNA expression, and epigenetic changes in a high-resolution, multi-dimensional manner. This review therefore provides an overview of results from recent genetic analyses and focuses on recent findings in complex multigenic etiology and epigenomic and transcriptional dynamics during cardiac reprogramming.

## 2. Epidemiology and Risk Factors for CHD

### 2.1. Epidemiology: Prevalence and Comorbidities

The individual lesions within CHD each feature their own prevalence, and the overall prevalence across all lesions in CHD is 1% among livebirths and 10% among stillbirths [28]. Isolated septal defects are the most common CHD lesions, with VSDs featuring an estimated prevalence of 3570 per million births and ASDs affecting 941 per million births.

Treatment of CHD involves procedural interventions in one-quarter of affected newborns during the first year of life [28]. The first year of life also features the majority of fatalities attributable to CHD, and the probability of surviving into adulthood increases to at least 75% after age one [29]. CHD is becoming increasingly prevalent in adults due to surgical improvements, and adults already outstrip the number of children living with the disease [1].

Increased survival of CHD patients into adulthood has revealed associated long term health risks that include increased risks for cancer, heart failure, and fatal arrythmias. CHD patients tend to experience higher rates of adverse health events compared to similarly aged individuals in the general population [29]. Some of these events, such as reversal of left-to-right shunts with Eisenmenger syndrome, are directly predicted by principles of physiology. Other adverse health events in CHD are likely linked to the genetic etiology, such as the elevated lifetime risk of cancer among CHD patients [15]. CHD patients whose disease occurs with additional phenotypes such as neurodevelopmental disabilities (NDD) or extracardiac abnormalities (ECA), or in whom the CHD is part of a known syndrome, tend to feature distinct genetics compared with patients with isolated CHD [17].

### 2.2. Non-Genetic Risk Factors: Maternal Exposures, Illnesses, Infectious Agents

The etiology of CHD is heterogeneous, and although the precise environmental contribution to CHD is unknown, environmental factors may contribute to an estimated 10% of CHD. These include, but are not limited to, pre-gestational diabetes, early onset pre-eclampsia, maternal obesity, in-utero exposures to alcohol, maternal Rubella, several medications including aspirin and carbamazepine, and a range of possible environmental exposures [23,24,25] (Figure 1). These environmental risk factors could also modify genetic risk in genetically predisposed individuals. For a full discussion of the non-genetic causes of CHD, we refer interested readers to the detailed research and clinical reviews on the subject [25,26,27].

## 3. Genetic Risk Factors and Relevant Biological Pathways

### 3.1. Aneuploidies and Copy Number Variations

Karyotype approaches detect chromosomal alterations greater than 5–10 Mb and discovered the first aneuploidies with associated CHD, including trisomy 21 (atrioventricular canal defect), trisomy 18 (VSD and pulmonary stenosis), trisomy 13 (ASD, VSD, and TGA), Turner syndrome (VSD, Aortic coarctation, and aortic stenosis), and Klinefelter syndrome (Ebstein anomaly and Tetralogy of Fallot [TOF]). Costain, Silversides, and Bassett briefly reviewed these aneuploidies and their relationships with CHD [30].

Copy number gains and losses are a considerable contributor to both syndromic and non-syndromic CHD. CHD frequently caused by copy number variations (CNVs) include 22q11.2 deletion syndrome, Williams–Beuren syndrome (7q11.23 deletion), Cri-Du-Chat syndrome (5p15.2 deletion), Cat eye syndrome (22q11 inversion or duplication), Jacobsen syndrome (11q deletion), 1p36 deletion syndrome, 1q21.1 deletion/duplication syndrome, 8p23.1 deletion syndrome, etc. [28,31]. The comprehensive summary of CNVs in CHD can be found in Fahed et al., Pierpont et al., and Costain et al. [28,30,31]. Large cohort-wide analysis of 538 CHD trios with whole-exome sequencing (WES) and genotype array revealed de novo CNVs in 9.8% patients without a previously identified genetic pathogenesis [18]. A recent study in an expanded cohort of 2517 CHD patients with WES identified de novo CNVs in 5.22% of cases, and the presence of de novo CNVs is also significantly associated with extracardiac anomalies and clinical outcomes such as transplant-free survival and time to final extubation [12].

### 3.2. De Novo Mutations

The first application of WES to systematically assess the impact of de novo single nucleotide variants and small insertions/deletions on CHD sequenced 362 trios (CHD patients and their parents) and used a case-control setup to show that CHD patients are relatively enriched for de novo mutations (DNMs) in genes highly expressed in the developing heart (HHE genes) [8]. Additionally, Zaidi et al. showed that CHD patients are enriched for DNMs in chromatin modifying genes; today, these are expected to account for 2.3% of all CHD. Later studies by Homsy and Jin would go on to show that chromatin modifying DNMs account for 28% of syndromic CHD (associated with NDD or ECA) [9,10].

The Zaidi study suggested that DNMs explain ~10% of all CHD, and Monte-Carlo simulations estimate that at least 400 genes enriched for pathogenic DNMs contribute to CHD [8,9,10]. However, the suite of DNM enriched CHD genes remains incomplete. The number of discovered CHD genes harboring more than one protein-damaging DNM grew from two in the Zaidi study (362 case trios and 264 control trios), to 21 in the Homsy study (1213 case trios), to 66 in the Jin study (2645 case trios), which clearly suggests that more CHD risk genes remain to be discovered [8,9,10].

**Figure 2:**
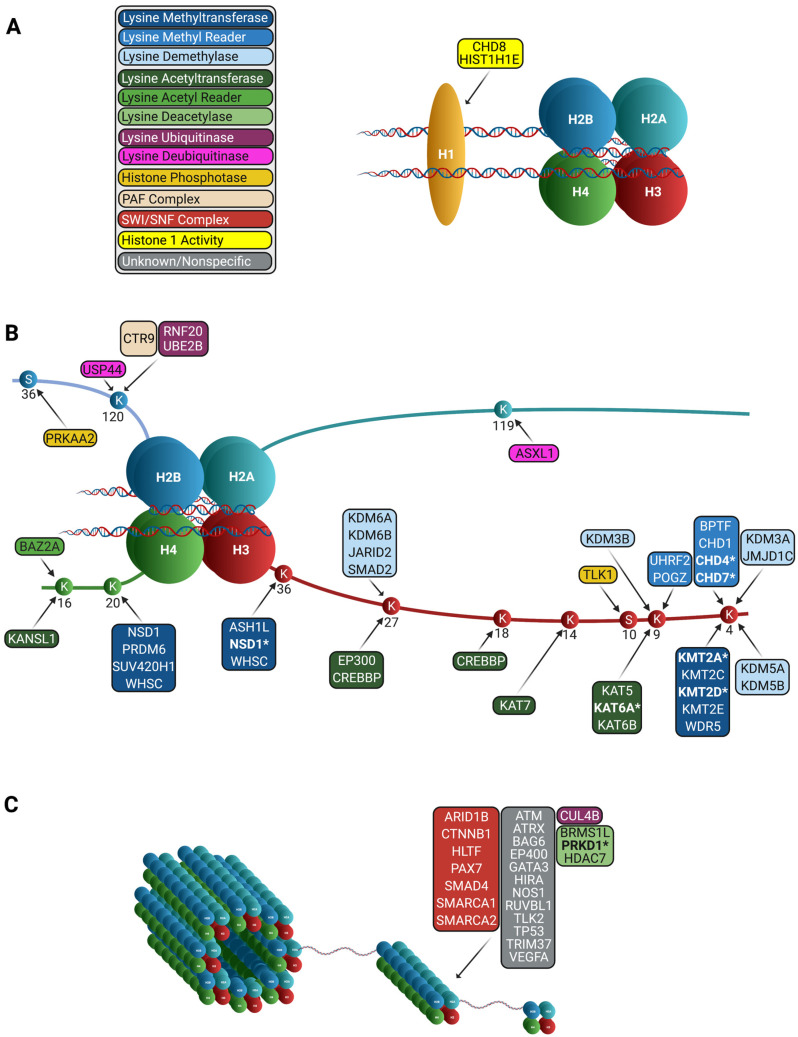
Chromatin remodeling genes in CHD. Genes denoted with an asterisk (*) are statistically significant. (**A**) Chromatin remodeling genes involved in CHD with H1 activity. (**B**) Chromatin remodeling genes involved in CHD with specified activity in the histone octamer. (**C**) Chromatin remodeling genes involved in CHD with unknown or nonspecific activities.

Findings from these studies posit that the most significant class of genes involved in CHD from DNMs are chromatin modifiers. Chromatin modifiers regulate transcription by controlling access to the DNA. Chromatin modifications, such as acetylation, methylation, and ubiquitination, occur on the four core histones (H2A, H2B, H3, and H4) (Figure 2). Since the addition and removal of chromatin modifications change DNA compactness, proper control during development is critical. For reviews on the topic of epigenetics and heart development, see reviews [32,33]. Given the major role of chromatin modifiers in heart development [34], it is not surprising that there is a significant enrichment in variants in chromatin modifiers in CHD patients compared to controls [35]. Variants in epigenetic modifiers can have pleiotropic effects on patients, since they affect the expression of many genes. Notably, patients with DNMs in chromatin modifiers have been shown to have increased risk of NDDs [9]. Mutations in these genes thus have the potential to be putative biomarkers predictive of clinical outcome in CHD.

Epigenetic marks change throughout heart development, and coincide with changes in gene expression, providing further evidence that their precise regulation is important [36]. Further, when these histone modifiers are knocked out in model organisms, heart abnormalities result. Morpholino knockdown of *kmt2d* (a H3K4 methyltransferase) in zebrafish results in heart morphology and looping that is abnormal [37]. KMT2D was shown to be important for depositing these marks on ion transport and cell cycle regulation genes in cardiomyocytes [38]. DOT1L (H3K79 methyltransferase) is responsible for pre-marking genes essential for cardiac commitment, such as *Tnni3* [39]. If HDAC1 and HDAC2 (histone deacetylases) are conditionally knocked out in the heart, cardiac morphological defects, cardiac arrhythmias, and cardiac stress occur [40]. The RNF20 complex (responsible for monoubiquitination of H2B) is important for regulating cardiac situs through modulating cilia gene expression [35]. Together, these studies demonstrate the role of epigenetic mechanisms in the transcriptional regulation is essential to left-right patterning and cardiac development.

### 3.3. Transmitted Mutations

It was not until recently that the role of transmitted mutations—a mechanism that had not been systematically studied due to a lack of sufficiently large cohorts and the absence of robust statistical methods—was shown to be accountable for a sizable proportion of CHD patients. The Jin study was the first to investigate transmitted mutations in a cohort of whole-exome sequenced CHD patients. This study developed a control-free regression-based model to estimate the expected number of recessive or dominant mutations and applied this model to 2871 CHD patients (including 2645 trios), ultimately finding that ~1.8% of cases are attributable to rare, transmitted mutations [10]. Among the most interesting are a founder mutation in *GDF1* accounting for 5% of CHD in Ashkenazim, recessive genotypes in *MYH6* in 11% of Shone complex probands, and loss-of-function mutations in *FLT4* accounting for 2% of TOF. Importantly, the link between *FLT4* loss-of-function mutations and TOF was later replicated by three independent studies [41,42,43], providing strong evidence of the involvement of vascular endothelial-derived growth factor (VEGF) signaling in the pathogenesis of TOF.

### 3.4. Biological Pathways

Unbiased genetic analyses in population scale CHD cohorts unequivocally support the role of multiple pathways in CHD pathogenesis, including cilia, Notch, VEGF, TGF-β, Wnt, Hedgehog, Ras, Nfatc1, and chromatin modifications [44,45]. Several major signaling pathways are summarized below.

Cilia, hair-like microtubule organelles that extend from the surface of mammalian cells, play an essential role during embryonic development, especially in determining left-right symmetry of the body [46]. Li et al. [47] identified 61 CHD recessive genes from a global mouse mutagenesis screen and consistently found half of them to be involved in cilia-related structures or pathways, such as IFT transport, cilia assembly, ciliary pocket, basal body, outer dynein arm, inversin compartment and transition zone, SHH signaling, WNT/planar cell polarity signaling, TGF-β/BMP signaling, and calcium signaling [47]. It thus suggests that cilia dysfunction can break symmetry at the organizer region or impact the asymmetric signal transduction through the lateral plate mesoderm to the developing heart to cause CHD [48,49]. In mice and humans, mutations in cilia-related genes typically lead to laterality defect, such as heterotaxy syndrome [47,50]. However, as cilia dysfunction often disrupts normal left-right patterning in a stochastic manner, and cilia are found within the developing heart itself, a wide range of phenotypes, including defects in atrioventricular septation, outflow tract, and valves, were also commonly observed [51].

The Notch signaling pathway is highly conserved in mammals and has various functions in cell development and differentiation, which plays an important role in the development of embryonic structures and organs, including the heart [52]. Notch signaling regulates heart development in many ways, such as heart field specification and heart looping [53]. Studies have showed that the variants in Notch pathway genes can cause CHD. Mutations in *NOTCH1* can cause heart lesions and either isolated or syndromic CHD. Additionally, it has been reported that CHD is present in almost all Alagille syndrome cases due to mutations in *NOTCH2* and its cognate ligand *JAG1*, where patients had minor valve defects to major structural malformations [54].

The VEGF signaling pathway, which is important in vasculogenesis and angiogenesis, has also been linked to CHD, predominantly in TOF [10,41,55,56]. The VEGF-related proteins (VEGF-A, VEGF-B, VEGF-C, and VEGF-D) and placenta growth factor comprise the VEGF family, which stimulate relative signaling by binding to specific tyrosine kinase receptors [57]. In human TOF, the enhanced myocardial VEGF expression could stunt angiogenesis [58]. Interestingly, mutations that significantly linked to risk for TOF were loss-of-function mutations in *FLT4* and *KDR* (Vegf receptor 2), which suggests deficient VEGF signaling as a plausible mechanism of TOF and related cardiovascular defects [41].

## 4. Genetic Modifiers and Complex Genetic Inheritance

Recent application of WES to identify monogenic causes for CHD has only led to molecular diagnoses for 20–30% of CHD patients [8,9,10,11,17,18]. Further, some families with presumed disease-causing genes (e.g., *NOTCH1*, *FLT4,* and *SMAD6*) display an inheritance pattern characterized by incomplete penetrance [10]. These findings highlight the genetic heterogeneity and complexity of CHD. It is plausible that genetic or environment modifiers could account for clinical variabilities in CHD presentation. Alternatively, more complex genetic models could underlie a significant proportion of CHD patients and awaits discoveries using improved genomic technologies and statistical models.

Genome-wide association studies (GWASs) have led to discoveries of many susceptibility loci as well as genetic modifiers for many complex human disorders [59], including CHD. These CHD GWASs have identified common genetic risk variants in multiple loci for atrial/ventricular septal defect [60,61,62,63], conotruncal heart defect [64], TOF [65], non-syndromic coarctation of the aorta [66], aortic valve stenosis [63,67], and hypoplastic left heart syndrome [64]. Although an independent replication phase was conducted for each reported significant genetic association, none of them could be reproduced across studies. Potential explanations about this irreproducibility include the difference in the ethnicity and/or the difference in CHD subtypes across studies, which should be carefully considered in the study design of future CHD GWAS. Further, the cohort size of these CHD GWASs is small compared to GWASs for other complex diseases [59,68,69,70]. No studies have been conducted with a focus on the identification of genetic modifiers for rare, damaging variants identified via WES and will be subjects for future studies. Finally, most of these GWASs only studied patients of European origin; genetic studies of multi-ethnic, diverse populations could improve discover for this heterogenous disease and reduce health disparities.

Besides GWAS, whole-genome sequencing has equipped molecular geneticists with the tools needed to further decipher the genetic agents of rare and complex diseases. As of today, only one large-scale whole-genome sequencing study has been performed, which used 763 CHD probands that had negative findings from WES and corresponding family members [16]. Although this study implicates enrichment of non-coding de novo mutations in CHD, the effect of such variants in CHD risk is comparably smaller than coding de novo or transmitted mutations [16]. This study estimates that the fraction of CHD cases that could be attributable to potentially disruptive non-coding functional variants is similar to the fraction of CHD cases that could be attributable to coding damaging de novo mutations; however, the actual contribution of non-coding de novo mutations as well as the investigation of complex genetic models (e.g., gene-gene and gene environment interaction) requires larger WGS cohorts to evaluate. It would be imperative to establish the molecular and cellular mechanisms associated with these non-coding regulatory elements, the transcriptional effect size, and their ability to alter heart development individually and synergistically with other relevant factors.

Since monogenic causes, aneuploidies, and known disease-causing CNVs cannot explain the majority of patients with congenital disorders, it is plausible that complex inheritance models such as epistatic effects of non-coding and coding variants can explain a proportion of the missing heritability. Like many other studies which investigated families with a sign of incomplete penetrance to find genetic modifiers, a recent study has presented a nuclear family in which three missense single-nucleotide variants in *MKL2*, *MYH7*, and *NKX2-5* are required to cause CHD [19]. Another study showed that interaction between DNAH6 and other primary ciliary dyskinesia genes led to heterotaxy [71]. Alternatively, the severity or manifestation of clinical conditions could be associated with the proportion of abnormal cell numbers in the disease-relevant tissues (i.e., high mosaicism rates are associated with severe disease phenotypes, whereas low-level mosaicism is generally observed in milder disease phenotypes or in presumably unaffected individuals) [72]. Although one recent study suggests that mosaic variants predicted as damaging had higher variant allele fraction than those predicted as benign in CHD patients [14], another study finds that mosaic variants do not account for a significant portion of CHD cases [73]. However, these two studies are limited by their small sample size. Continued sequencing of large, well-phenotyped cohort will provide an increasingly complete picture of genetic underpinning of CHD.

## 5. Discussion

Recent studies have significantly increased our understanding of various CHD genetic etiologies, associated molecular pathways, and potential roles for complex genetics in CHD. One unambiguous conclusion from these studies was that we have not identified even half of the genes making up the 10% of CHD caused by pathogenic DNMs. This implies that some fraction of genetically unsolved CHD cases feature unrecognized pathogenic DNMs. The same logic applies to transmitted dominant and recessive mutations. The set of known de novo or transmitted genetic variants contributing to CHD is expected to increase over the next several years as increasingly large CHD cohorts are recruited and analyzed. Further, we expect that targeted resequencing of compelling genes arising from these studies will allow efficient and scalable probing of hundreds of genes in thousands of individuals. This will provide a well-powered cohort to implicate novel CHD risk genes among the targeted gene panel and have near-term clinical impact.

Even as monogenic etiologies of CHD remain incompletely understood, studies focused on the complex genetic architecture of CHD have much to offer in the way of potential molecular diagnoses. Polygenic risk scores (PRS) are a tool initially developed to quantify genetic risk for common diseases such as coronary artery disease, psychiatric illness, and various types of cancers [74]. For diseases such as coronary artery disease, PRS have been shown to predict those patients whose genetic disease risk is greater than or equal to the risk conferred by known monogenic lesions in coronary artery disease genes [75]. For developmental diseases such as CHD, whose presence or absence can typically be ascertained at birth, the clinical utility of PRS lies not in predicting genetic risk but in potentially offering insight into the genetic etiology of patients whose disease cannot be explained by monogenic causes. Although the application of PRS to CHD is in its infancy [76], we expect in the near future that PRS will be used to provide accurate risk assessment for developing diseases that are more prevalent in CHD patients such as NDD and cancer, and to examine environmental interactions with potential genetic factors.

Of note, development of PRS typically requires genotyping of cohorts substantially larger than what is currently available for CHD. PRS developed for coronary artery disease, psychiatric illness, and various cancers employed population scale cohorts such as the UK Biobank [75]. PRS in CHD have been developed with smaller cohorts of CHD patients with Down Syndrome [76]. The application of PRS to CHD could be directed towards exploring the complex genetic basis for phenotypic variability in patients with a shared established lesion (e.g., Down Syndrome or 22q11 syndrome), or could be more generally used to understand polygenic risk in CHD (by applying PRS in collections of CHD patients without regards to a specific genotype or CHD subtype). Importantly, the accuracy of PRS is thought to be specific to the population in which PRS was developed, making the potential application of CHD PRS to diverse communities an anticipated challenge.

Because as many as ~55% of CHD cases may not have a readily identified etiology, there is still much work to be done to reveal a complete picture of etiologies involved in CHD. It is highly likely that additional molecular diagnoses may be discovered by prioritizing diverse patient recruitment or by focusing on population-specific CHD genotypes. CHD, like other developmental diseases, may feature genetic etiologies that are unique between ancestrally diverse groups. Further, functional genomic studies using single-cell RNA-sequencing, ChIP-seq, and ATAC-seq have started and will reveal complex, intrinsic genetic networks with effects in specific cardiac lineages in early heart development [77,78,79,80]. Analysis of increasingly diverse CHD cohorts is ultimately likely to reveal hitherto unknown monogenic causes of CHD. In vitro/in vivo functional validations linked with patients’ phenotypic data will provide clinicians with resources to apply human genetic findings toward the clinical care of CHD patients.
